# De-Palmitoylation of Tissue Factor Regulates Its Activity, Phosphorylation and Cellular Functions

**DOI:** 10.3390/cancers13153837

**Published:** 2021-07-30

**Authors:** Camille Ettelaie, Sophie Featherby, Araci M. R. Rondon, John Greenman, Henri H. Versteeg, Anthony Maraveyas

**Affiliations:** 1 Biomedical Section, University of Hull, Cottingham Road, Hull HU6 7RX, UK; S.Featherby-2016@hull.ac.uk (S.F.); j.greenman@hull.ac.uk (J.G.); 2 Einthoven Laboratory for Vascular and Regenerative Medicine, Division of Thrombosis and Hemostasis, Department of Internal Medicine, Leiden University Medical Center, 2333 ZA Leiden, The Netherlands; A.M.da_Rocha_Rondon@lumc.nl (A.M.R.R.); H.H.Versteeg@lumc.nl (H.H.V.); 3 Division of Cancer-Hull York Medical School, University of Hull, Cottingham Road, Hull HU6 7RX, UK; anthony.maraveyas@hey.nhs.uk

**Keywords:** tissue factor, factor VIIa, encryption, palmitoylation, transmembrane-domain, palmitoyl-protein thioesterase

## Abstract

**Simple Summary:**

The relationship between cancer and blood clotting has been well established. The activation of blood coagulation proteins regulates the fate of cells and is known to be used by cancer cells to enhance survival and proliferation. Cells strictly regulate the initiation of coagulation through controlling the action of the protein “tissue factor (TF)”. In addition to initiating clotting, TF also acts as a deciding factor to determine the extent of damage and instructs cells to proliferate and repair or, when severely damaged, to die. Therefore, normal cells keep TF in a dormant state, achieved through mechanisms called “TF encryption”. Understanding the mechanisms by which the cells control the activity of TF is crucial, especially since cancer cells bypass these regulatory mechanisms, ensuring survival and tumour growth. This study has elucidated essential molecular mechanisms by which cells regulate TF clotting activity, and also the cellular signals arising from these.

**Abstract:**

In this study, the role of de-palmitoylation of tissue factor (TF) in the decryption of its activity was explored. TF-tGFP constructs were prepared by mutagenesis-substitution at Cys245 to prevent or mimic palmitolyation. Additionally, to reduce TF de-palmitoylation, the expression of palmitoyl-protein thioesterases (PPT) was suppressed. Other TF mutants were prepared with altered flexibility, hydrophobicity or length of the transmembrane domain. The outcome of these alterations on fXa-generation, fVIIa binding, Ser253 phosphorylation and TF-microvesicle release were assessed in endothelial cells, and the influence on endothelial and MCF-7 cell proliferation and apoptosis was analysed. Preventing TF palmitoylation (TF_Ser245_-tGFP), increasing the hydrophobicity (TF_Phe241_-tGFP) or lengthening (TF_LongTM_-tGFP) of the transmembrane domain enhanced fXa-generation in resting cells compared to cells expressing TF_Wt_-tGFP, but fXa-generation was not further increased following PAR2 activation. Extending the available length of the transmembrane domain enhanced the TF-tGFP release within microvesicles and Ser253 phosphorylation and increased cell proliferation. Moreover, prevention of PKCα-mediated Ser253 phosphorylation with Gö6976 did not preclude fXa-generation. Conversely, reducing the hydrophobicity (TF_Ser242_-tGFP), shortening (TF_ShortTM_-tGFP) or reducing the flexibility (TF_Val225_-tGFP) of the transmembrane domain suppressed fXa-generation, fVIIa-HRP binding and Ser253 phosphorylation following PAR2 activation. PPT knock-down or mimicking palmitoylation (TF_Phe245_-tGFP) reduced fXa-generation without affecting fVIIa binding. This study has for the first time shown that TF procoagulant activity is regulated through de-palmitoylation, which alters the orientation of its transmembrane domain and is independent of TF phosphorylation. However, Ser253 phosphorylation is facilitated by changes in the orientation of the transmembrane domain and can induce TF-cellular signalling that influences cellular proliferation/apoptosis.

## 1. Introduction

Uncontrolled coagulation arising from tissue factor (TF) activity has been implicated in both arterial and venous thrombosis with potentially fatal outcomes [1,2]. TF encryption refers to the post-translational suppression of TF procoagulant activity on the surface of cells. Unperturbed or non-activated cells present little TF activity despite the presence of TF antigen on the cell surface. Interestingly, encrypted TF may form a complex with fVIIa without promoting significant coagulation, and the kinetics of this interaction have previously been studied [3]. However, it is suggested that the affinity between these two proteins is amplified following decryption. The mechanism of TF decryption is not currently understood, although at least four mechanisms have been suggested [4,5,6,7,8]. However, none of these alone is sufficient to explain the observed tight regulation of TF activity.

TF has a small cytoplasmic domain that has no kinase activity and is not thought to be required for its procoagulant activity [9,10]. However, Ser253 and Ser258 within the cytoplasmic domain of TF are phosphorylated following de-palmitoylation of the cytoplasmic domain [11,12] and are capable of regulating the incorporation of TF into microvesicles [13]. The role of phosphorylation in the regulation of incorporation of TF into microvesicles has been characterised [13,14,15,16,17]. Phosphorylation of Ser253 permits the interaction of TF with the cytoskeletal protein filamin-A, which is required for the trafficking of TF to the site of microvesicle formation [14,15,16]. Palmitoylation of Cys245 within the cytoplasmic domain of human TF was one of the earliest post-translational modifications reported [18] and was suggested to be a means of anchoring TF in cholesterol-rich lipid rafts [5,6] containing procoagulant phospholipids [4,11,19]. However, experimental data indicated that the procoagulant activity of TF is reduced following palmitoylation [11,12,19]. It has also been shown that de-palmitoylation of TF enhances and precedes TF phosphorylation [11]. We have recently shown that subsequent TF phosphorylation at Ser253 results in increased TF procoagulant activity [14] as well as TF release within cell-derived microvesicles [13]. Other studies have shown that the association of TF with lipid-raft occurs as part of the mechanism of TF release into microvesicles [14,20] and control of TF procoagulant activity [21,22,23]. In this study, we hypothesised that the de-palmitoylation of TF alters the orientation of the transmembrane domain of TF within the plasma membrane. This in turn could permit the transfer and association of TF with thicker membrane regions such as cholesterol-rich microdomains, which contain lipids that support TF activity. We also explored the association of these events with TF-mediated proliferative and pro-apoptotic signals.

## 2. Material and Methods

### 2.1. Site-Directed Mutagenesis

The pCMV6-Ac-TF-tGFP plasmid DNA (OriGene/Insight Biotechnology, Wembley, UK) was used to express wild-type or variant forms of TF, as previously described [13,24,25]. Preparations of pCMV6-Ac-tGFP (control) and pCMV6-Ac-TF_Ala253_-tGFP were described previously [13]. Single amino acid-substitutions or deletions were carried out to obtain the mutants, as illustrated in Table 1, using the primer pairs indicated. All procedures were carried out using the Q5-site directed mutagenesis kit (New England Biolabs, Hitchin, UK) according to the manufacturer’s procedure. All mutants were verified by sequencing (Eurofin MWG, Wolverhampton, UK).

### 2.2. Cell Culture, Transfection and Cellular Activation

Human dermal blood primary endothelial cells (HDBEC) and coronary artery endothelial cells (HCAEC) devoid of endogenous TF were cultured in MV media containing 5% (*v*/*v*) foetal calf serum (FCS) and growth supplements (PromoCell, Heidelberg, Germany). MCF-7 and MDA-MB-231 breast cancer cell lines (ATCC, Teddington, UK) expressing low and high levels of TF were cultured in EMEM and DMEM, respectively, containing 10% (*v*/*v*) FCS. Cells (5 × 10^5^) were seeded out into 48-well plates and transfected with 0.5 µg of pCMV6-Ac-TF-tGFP plasmid DNA or, alternatively, mutant forms of the plasmid, as shown in Table 1. Transfection of the cells was carried out using TransIT-2020 (Geneflow, Lichfield, UK) according to the manufacturer’s instructions. Cells were permitted to express the proteins for 48 h, and the expression of the TF variants was confirmed by flow cytometry as before [13,14]. Prior to experiments, cells were pre-adapted to respective serum-free media for 1 h and then activated by incubation with protease-activated receptor 2-activating peptide (PAR2-AP); SLIGKV; (20 µM). In some experiments, cells were transfected with a specific set of SilencerSelect^®^-siRNA (10 pmol; Life Technologies, Paisley, UK) to suppress the expression of palmitoyl–protein thioesterase (PPT) 1 and 2 concurrently, or transfected with a comparable set of control siRNA (10 pmol; Life Technologies) for 48 h prior to activation. The concentration of siRNA to knock-down PPT1 and PPT2 expression was optimised by western blot. To block the phosphorylation of TF at Ser253, cells were pre-incubated with the PKC inhibitor Gö6976 (100 nM; R&D Systems, Abingdon, UK) for 40 min, prior to activation.

### 2.3. Cell Proliferation and Apoptosis Assays

Cell numbers were determined by staining with crystal violet as previously described [26,27] and calculated from a standard curve. In addition, cellular apoptosis was quantified using the TiterTACS™ Colorimetric Apoptosis Detection Kit (AMS Biotechnology, Abingdon, UK) according to the manufacturer’s instructions [28,29].

### 2.4. Determination of Microvesicle Density and TF Antigen

The release of microvesicles was examined using the Zymuphen MP-assay kit (Hyphen BioMed/Quadratech, Epsom, UK) and the microvesicle density was determined from the standards provided. The properties of the MV were previously confirmed [29,30]. The released microvesicle-associated TF antigen was measured using the Quantikine TF-ELISA kit (R & D Systems) according to the manufacturer’s instructions. Cell surface TF antigen was measured in situ using an ELISA-based procedure as previously described [15,24].

### 2.5. Cell-Based Factor Xa-Generation Assay

Cell surface TF-fVIIa activity was measured by modification of previously described procedures [3,24,31]. Cells (5 × 10^4^) were seeded out into 48-well plates and transfected with the appropriate wild-type or mutant TF plasmids, as shown in Table 1. The cells were washed with phosphate-buffered saline (PBS) pH 7.4 and then pre-adapted to serum-free medium. The cells were incubated with PAR2-AP for up to 60 min and then washed and incubated with fVIIa (20 nM; Enzyme Research Labs, Swansea, UK) in HEPES-buffered saline (HBS) pH 7.4, containing 1% (*w*/*v*) bovine serum albumin (BSA) and 5 mM CaCl_2_ (100 µL) for an additional 10 min. Finally, the samples were supplemented with fX (100 nM) together with fXa substrate (0.2 mM; Hyphen) diluted in the same buffer (100 µL). The samples were incubated for 60 min to develop the colour. Aliquots (150 µL) were then transferred to a 96-well plate containing 2% (*v*/*v*) acetic acid (50 µL) and the absorptions were measured immediately at 405 nm. The amount of fXa generated was determined using a standard curve prepared using fXa (Enzyme Research Labs). To inhibit cell surface TF activity, cells were pre-incubated with the inhibitory anti-TF antibody HTF1 (40 µg/mL; eBioscience/Thermo Scientific, Warrington, UK) prior to the addition of fVIIa [15].

### 2.6. Factor VIIa Binding Assay

In order to measure the binding of fVIIa to cell-surface TF, fVIIa (30 µg) was conjugated to horseradish peroxidase (HRP) using the Innova Lightning-link HRP kit (Expedeon, Cambridge, UK). A similar control was also prepared by conjugating BSA (30 µg) to HRP. After neutralisation of excess HRP, the specificity of the fVIIa to interact with TF was confirmed as follows; 96-well plates were coated with recombinant Innovin TF (13 ng/mL; Dade Behring, Deerfield, MA, USA) in PBS containing BSA (1% *w*/*v*), or coated with the vehicle solution alone by incubating overnight at 4 °C. In parallel experiments, sets of endothelial cells (5 × 10^4^) were plated out into 48-well plates and transfected to express TF-tGFP or tGFP, along with non-transfected cells. All plates were washed with PBS and incubated with fVIIa-HRP (20 nM) (100 µL) for 15 min. The plates were then washed three times with PBS, and HRP activity was determined using the TMB One-solution HRP substrate (200 µL). Aliquots (150 µL) of the substrate were then transferred into fresh wells containing 2 M H_2_SO_4_ (50 µL), and the absorptions were determined at 450 nm. The amount of bound fVIIa-HRP was determined from a standard curve prepared with a range of concentrations of fVIIa-HRP (100 µL). To confirm the fVIIa activity of the HRP-conjugated preparations, fXa-generation potential was determined on both endothelial cells transfected to express TF and also immobilised-recombinant TF using a range of fVIIa-HRP concentrations (0–10 nM) and compared to fXa generation using unlabelled-fVIIa.

### 2.7. Immunoprecipitation of TF and Western Blot Analysis of TF Phosphorylation

TF-tGFP was immunoprecipitated from cells lysed in PhosphoSafe buffer (Novagen/Merck Millipore, Watford, UK) using anti-tGFP-magnetic beads (clone 2H8) (25 μL, OriGene/Insight Biotechnology) or, in some cases, using anti-TF antibody (HTF1) followed by protein A-magnetic bead purification, as previously described [24,25]. The samples were separated by SDS-PAGE, transferred onto nitrocellulose membranes and probed with a rabbit anti-TF antibody (FL295) (Santa Cruz Biotechnology, Heidelberg, Germany) diluted 1:4000 (*v*/*v*) in TBST. The membranes were developed with goat anti-rabbit alkaline phosphatase-conjugated antibody (Santa Cruz), diluted 1:4000 (*v*/*v*), and bands were visualised using the Western Blue stabilised alkaline phosphatase-substrate (Promega, Southampton, UK). Phosphorylation of TF on Ser253 was detected in the immunoprecipitated samples using a rabbit anti-phospho-PKC-substrate motif antibody (Cell Signalling Technology) diluted 1:2000 (*v*/*v*) in TBST buffer and detected as above. Analysis of PPT1 and PPT2 expression in cell lysates with and without siRNA-mediated silencing of PPT was carried out using rabbit anti-human PPT1 and PPT2 antibodies (CloudClone/Insight Biotechnology, Wembley, UK) diluted 1:2000 (*v*/*v*) in TBST and detected as above.

### 2.8. Confocal Microscopy

HDBEC (5 × 10^4^) were seeded out into 35 mm glass-based μ-dishes (InVitro Scientific/Cellvis, Sunnyvale, CA, USA) and transfected to express wild-type or variants of TF-tGFP [14,31]. In some experiments, the cells were co-transfected with siRNA to silence the expression of PPT1 and PPT2, concurrently. Sets of cells were activated with PAR2-AP for 30 min and then fixed and labelled with DAPI (2 µg/mL) [14,31]. The samples were then analysed by confocal microscopy at room temperature using a Zeiss LSM 710 confocal microscope with a ×63 water immersion objective. Images were acquired using the ZEN software (Carl Zeiss Ltd., Welwyn Garden City, UK) and analysis of the aggregate sizes was carried out using the ImagePro Plus software (Media Cybernetics, Bethesda, MD, USA).

### 2.9. Statistical Analysis

All data represent the calculated mean values from the number of experiments stated in each figure legend ± the calculated standard error of the mean. Statistical analysis was carried out using the Statistical Package for the Social Sciences (SPSS Inc., Chicago, IL, USA). Significance was determined using one-way ANOVA (analysis of variance) and Tukey’s honest significance test or, where appropriate, by paired *t*-test.

## 3. Results

### 3.1. Validation of the fVIIa-HRP Conjugation and fVIIa Activity

Prior to usage, the ability of the fVIIa-HRP conjugate preparation to bind to recombinant TF (Supplementary Figure S1A) and cell surface TF_Wt_-tGFP (Supplementary Figure S1B) was confirmed in human primary endothelial cells. Additionally, fVIIa-HRP and non-conjugated fVIIa exhibited similar fXa-generation activities (Supplementary Figure S1C).

### 3.2. Non-Active/Encrypted TF Is Capable of Binding fVIIa without Increasing Procoagulant Activity

**Figure 1:**
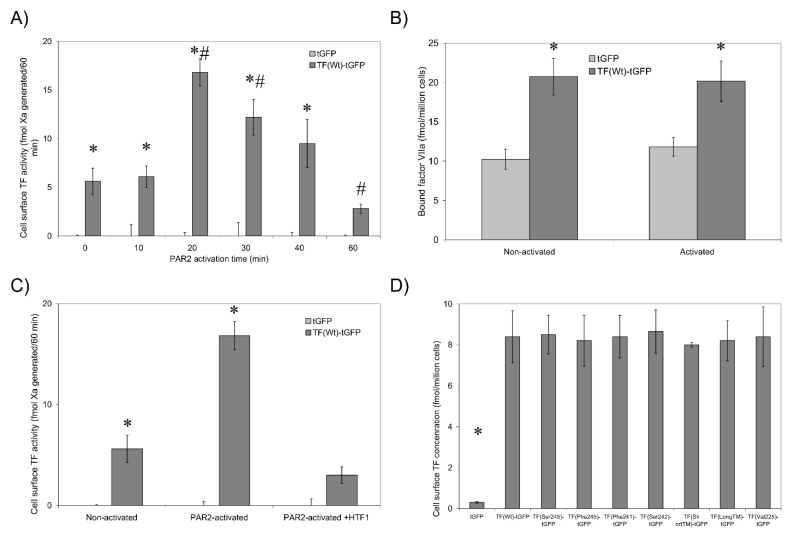
Analysis of TF-tGFP activity and fVIIa-HRP binding to TF. (**A**) The ability of HDBEC (5 × 10^4^) expressing either TF-tGFP or tGFP to support fXa-generation was measured before and after activation of the cells with PAR2-AP (20 µM) for up to 60 min. (*n* = 10, * = *p* < 0.05 vs. non-activated cells expressing tGFP; # = *p* < 0.05 TF_Wt_-tGFP samples vs. non-activated cells expressing TF_Wt_-tGFP.) (**B**) HDBEC were incubated with fVIIa-HRP (20 nM) for 10 min and washed, and the amount of bound fVII-HRP was determined using HRP substrate. (*n* = 3, * = *p* < 0.05 vs. respective cells expressing tGFP.) (**C**) HDBEC were pre-incubated with the inhibitory TF antibody HTF1 (40 µg/mL) prior to analysis using the fXa-generation assay. (*n* = 3, * = *p* < 0.05 vs. respective controls expressing tGFP.) (**D**) The expression of TF variants on the surface of the transfected HDBEC was quantified using an ELISA-based procedure. (*n* = 4, * = *p* < 0.05 vs. cells expressing TF_Wt_-tGFP.)

The activation of cells by incubation with the PAR2-agonist peptide is a reproducible procedure [13,14] that permits the accurate measurement of changes in fXa-generation in a short period, which was imperative to the success of this study. Non-activated cells transfected to express TF_Wt_-tGFP exhibited a low level of fXa-generation (Figure 1A). Furthermore, cells expressing tGFP (Figure 1A) or non-transfected cells (not shown) exhibited little fXa-generation potential. Activation of PAR2 resulted in increased fXa-generation in HDBEC expressing TF-tGFP, reaching a peak at around 20 min post-activation. The interaction between TF and fVIIa was robust, and the fXa-generation did not change by washing out excess fVIIa. Interestingly, no increase in cell-surface TF-tGFP antigen was observed in endothelial cells at 20 min post-activation (not shown). Moreover, fVIIa-HRP was capable of binding to cell surface TF-tGFP at similar magnitudes in both non-activated cells and following PAR2 activation for 20 min (Figure 1B and Table 2). Pre-incubation of cells with the inhibitory HTF1 antibody significantly reduced fXa-generation (Figure 1C), indicating the requirement for the TF-fVIIa complex. Together, these data indicate that the observed elevation in fXa-generation following cell activation was not due to change in the availability of TF for fVIIa binding. Furthermore, any variations in fXa-generation and fVIIa-HRP binding did not arise from differences in cell surface expression of the TF-tGFP variants, demonstrated by the comparable cell-surface TF antigen (Figure 1D).

### 3.3. De-Palmitoylation of Cys245 Is Required for TF-fVIIa Activity but Not Complex Formation

**Figure 2:**
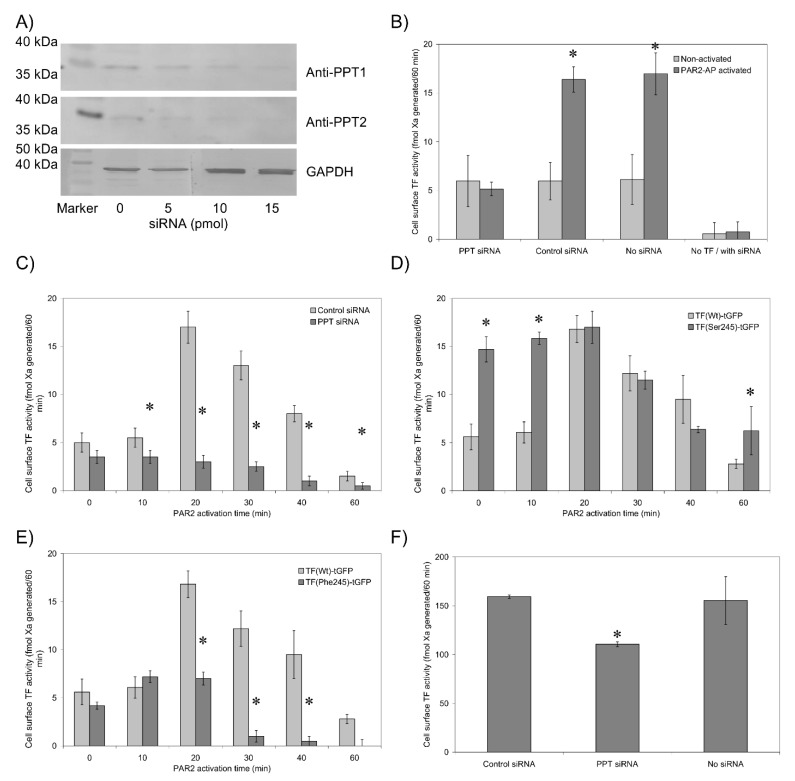
Analysis of TF-tGFP procoagulant activity and fVIIa-HRP binding in the presence and absence of TF palmitoylation. (**A**) HDBEC (5 × 10^4^) were transfected with PPT-siRNA (0–15 pmol) and the expression of PPT1 and -2 measured by western blot. (**B**) HDBEC were co-transfected with combinations of pCMV-Ac-TF-tGFP and PPT-siRNA or control siRNA, and fXa-generation was measured in resting and PAR2-activated cells. (*n* = 5, * = *p* < 0.05 vs. the respective non-activated sample.) (**C**) HDBEC were co-transfected to express TF-tGFP, with PPT siRNA or control siRNA. Factor Xa-generation was measured before and after PAR2 activation for up to 60 min. (*n* = 4, * = *p* < 0.05 vs. the respective control siRNA sample.) HDBEC were transfected to express (**D**) TF_Ser245_-tGFP or (**E**) TF_Phe245_-tGFP and fXa-generation were measured as above. (*n* = 6, * = *p* < 0.05 vs. the respective cells expressing TF_Wt_-tGFP.) (**F**) MDA-MB-231 (5 × 10^4^) cells expressing endogenous TF were transfected with PPT-siRNA or control siRNA and fXa-generation measured (*n* = 3, * = *p* < 0.05 vs. cells without siRNA).

Two approaches were employed in order to examine the role of de-palmitoylation in the decryption of TF activity. The only palmitoylation site on TF is at Cys245, and PPT1 and PPT2 are the two identified enzymes shown to be responsible for the de-palmitoylation of cellular proteins, although controversies exist regarding such proteins [32]. Therefore, siRNA-mediated knock-down of both PPT1 and PPT2 was optimised to reduce the expression of these two genes concurrently (Figure 2A). Analysis of PAR2-activated cells previously transfected to express TF_Wt_-tGFP, together with the PPT-siRNA, showed significantly reduced fXa-generation following PAR2 activation of HDBEC (Figure 2B,C) and HCAEC (Supplementary Figure S2) compared to cells transfected with control siRNA. However, the ability of fVIIa-HRP to interact with cell surface TF-tGFP remained unhindered by the suppression of PPT expression (Table 2). To confirm these data, the substitution of Cys245 with serine was carried out to prevent the palmitoylation of TF. Conversely, the substitution of this residue to phenylalanine was used to increase the hydrophobicity of the residue at position 245 and encourage its incorporation into the membrane. Prevention of TF palmitoylation by expressing TF_Ser245_-tGFP significantly increased fXa-generation in resting cells, compared to TF_Wt_-tGFP (Figure 2D and Supplementary Figure S2). However, the level of activities converged following activation of cells with PAR2-AP. The ability of fVIIa-HRP to bind to the cells remained unaffected by Cys245→Ser substitution (Table 2). In contrast, the expression of TF_Phe245_-tGFP prevented the increase in fXa-generation following cell activation but did not alter the residual activity in resting cells (Figure 2E). Additionally, the ability of fVIIa-HRP to interact with cell surface TF_Phe245_-tGFP was partially impaired following activation (Table 2). In agreement with the data generated with transfected-endothelial cells, siRNA-mediated knock-down of PPT1 and PPT2 in the breast cancer cell line MDA-MB-231, which constitutively expresses TF, also reduced fXa-generation (Figure 2F).

### 3.4. De-Palmitoylation of Cys245 Promotes the Aggregation of TF on the Cell Membrane

**Figure 3:**
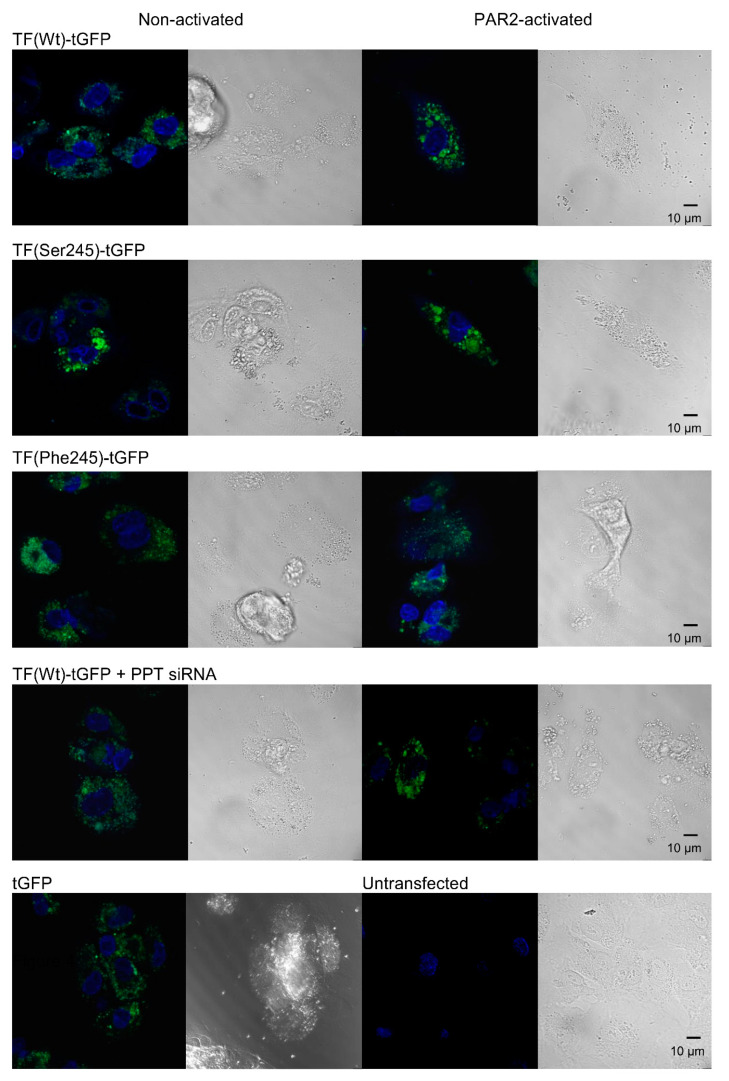
Confocal microscopy analysis of TF-tGFP distribution within cells. HDBEC (5 × 10^4^) were transfected to express TF_Wt_-tGFP in the presence and absence of PPT-siRNA or to express TF_Ser245_-tGFP or TF_Phe245_-tGFP. Sets of cells were then activated using PAR2-AP (20 µM), washed and fixed with 3% (*v*/*v*) formaldehyde. All cells were labelled with DAPI and analysed using a Zeiss LSM 710 confocal microscope.

It has been reported that the procoagulant activity of TF is strongly influenced by the membrane microenvironment [21,22,23]. We hypothesised that de-palmitoylation of TF following cellular activation may sequester TF molecules into specific microdomains within the cell membrane. Therefore, the aggregation of TF-tGFP variants before and after cellular activation was examined by confocal microscopy. These were compared to cells expressing TF_Wt_-tGFP in which the expression of PPT1 and PPT2 was silenced. Examination of the cells indicated an outspread cellular distribution of TF_Wt_-tGFP (Figure 3), which then aggregated following activation, forming regions with a higher mean diameter (Table 3). Interestingly, TF_Ser245_-tGFP aggregates were detected even prior to cell activation. In contrast, expression of TF_Phe245_-tGFP or suppression of the PPT expression reduced the ability of the TF protein to form aggregates following PAR2 activation.

### 3.5. TF Activity Is Regulated by the Length and Orientation of the Transmembrane Domain

**Figure 4:**
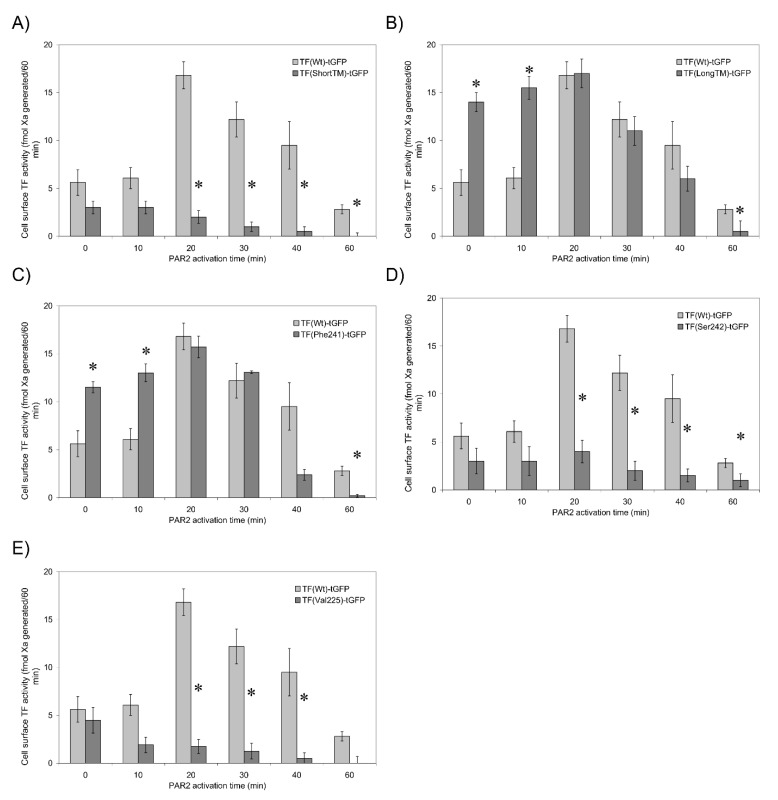
The influence of the modifications of TF transmembrane domain on TF activity. HDBEC (5 × 10^4^) were transfected to express (**A**) TF_ShortTM_-tGFP, (**B**) TF_LongTM_-tGFP, (**C**) TF_Phe241_-tGFP, (**D**) TF_Ser242_-tGFP or (**E**) TF_Val225_-tGFP, and fXa-generation was measured. (*n* = 4, * = *p* < 0.05 vs. the respective cells expressing TF_Wt_-tGFP.)

Palmitoylation can adjust the orientation of transmembrane domains within the membrane by altering the length of the transmembrane domain [33]. This can influence the location of the protein within the membrane [34,35,36]. The peptide at the cytoplasmic-transmembrane interface of TF consists of a sequence of amino acids, which renders two of the amino acids (Ser241-Leu242) a neutral overall hydrophobicity. We envisaged that these amino acids may be accommodated equally well within the aqueous cytoplasm and within the hydrophobic membrane. Consequently, conformational changes within TF, arising from de-palmitoylation of Cys245, would permit the Ser241-Leu242 motif to be incorporated into the membrane and therefore lengthen the transmembrane domain by two amino acids. Deletion of Ser241-Leu242 to shorten the transmembrane domain (TF_ShortTM_-tGFP) prevented the increase in fXa-generation following PAR2 activation (Figure 4A and Supplementary Figure S2). Interestingly, the interaction of fVIIa with TF_ShortTM_-tGFP only declined following cellular activation (Table 2). In contrast, duplicating the motif to lengthen the transmembrane domain (TF_LongTM_-tGFP) increased the fXa-generation potential in resting cells (Figure 4B and Supplementary Figure S2) but had no outcome on fVIIa binding (Table 2). By substituting Ser241→Phe or alternatively Leu242→Ser, it was possible to manipulate the hydrophobicity of this region and potentially increase or decrease the length of the transmembrane region, respectively. Using this approach, a marginally enhanced level of fXa-generation was detected in resting cells expressing TF_Phe241_-tGFP compared to cells expressing TF_WT_-tGFP, which increased to the same levels as that of wild-type form upon PAR2 activation (Figure 4C and Supplementary Figure S2). Moreover, the binding of fVIIa-HRP to TF was unaffected by Ser241→Phe substitution (Table 2). Basal fXa-generation activity in cells expressing TF_Ser242_-tGFP was comparable to that of TF_Wt_-tGFP but did not increase following cellular activation (Figure 4D and Supplementary Figure S2). However, the fVIIa-HRP binding remained unaffected by Leu242→Ser substitution (Table 2). Finally, the requirement for a flexible transmembrane domain, capable of accommodating different orientations of the protein, was explored by substituting Gly225 for a valine residue and therefore reducing the flexibility of the transmembrane domain. The expression of TF_Val225_-tGFP supported basal levels of fXa-generation in resting cells, but again, there was no enhancement in fXa-generation following PAR2 activation (Figure 4E), which was concurrent with a reduction in fVIIa-HRP binding ability (Table 2).

### 3.6. The Transmembrane Domain Regulates TF Phosphorylation at Ser253 and Incorporation into Microvesicles

**Figure 5:**
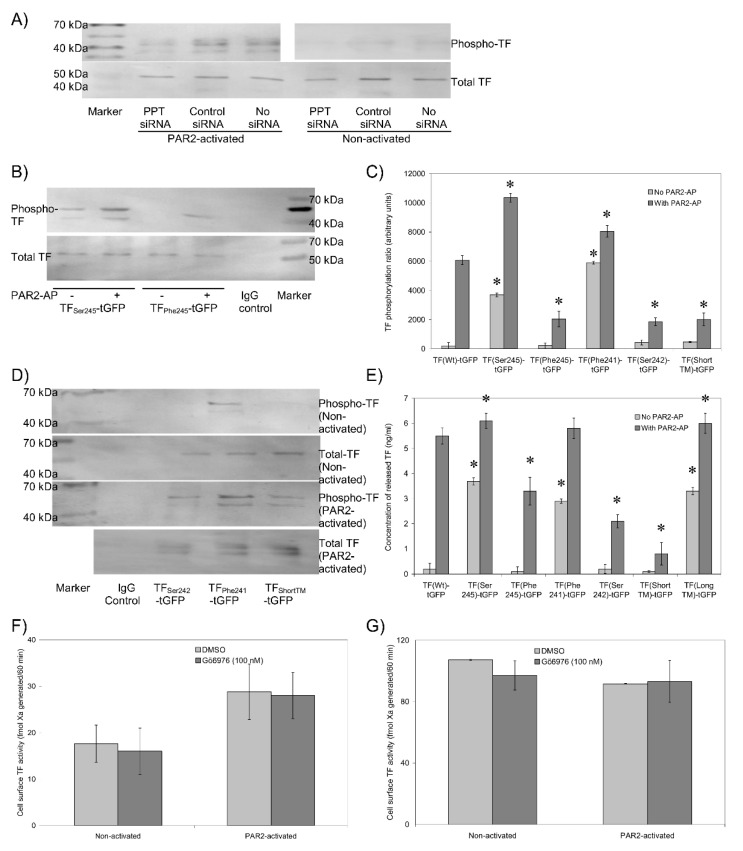
Analysis of Ser253 phosphorylation in the TF variants. HDBEC (2 × 10^5^) were transfected (**A**) to express TF_Wt_-tGFP in the presence and absence of PPT-siRNA or (**B**) to express TF_Ser245_-tGFP or TF_Phe245_-tGFP. Cells were activated with PAR2-AP (20 µM) for 20 min, and TF-tGFP was then immunoprecipitated and analysed by western blot and (**C**) the phosphorylation of Ser253 was quantified. (*n* = 3, * = *p* < 0.05 vs. the respective cells expressing TF_Wt_-tGFP.) (**D**) HDBEC (2 × 10^5^) were transfected to express TF_Phe241_-tGFP, TF_Ser242_-tGFP, TF_ShortTM_-tGFP or TF_Val225_-tGFP, and the phosphorylation of Ser253 was analysed and (**C**) quantified. (**E**) HDBEC (5 × 10^4^) were transfected with the TF variants as shown, and the concentration of TF released from cells was measured by TF-ELISA. (*n* = 4, * = *p* < 0.05 vs. the respective cells expressing TF_Wt_-tGFP.) (**F**) HDBEC (5 × 10^4^) were transfected to express TF_Wt_-tGFP and pre-incubated with Gö6976 (100 nM) prior to PAR2-activation and fXa-generation measured in resting and activated cells. (*n* = 4). (**G**) MDA-MB-231 (5 × 10^4^) cells expressing endogenous TF were incubated with Gö6976 and fXa-generation measured (*n* = 3).

De-palmitoylation of TF precedes its phosphorylation at Ser253 [11], which in turn is required for the incorporation of TF into microvesicles [13,15]. Therefore, an attempt was made to examine the outcome of TF de-palmitoylation and the change in the transmembrane domain on the phosphorylation of TF. Cells were transfected with PPT-siRNA together with TF_Wt_-tGFP or the mutant forms of TF-tGFP described above. TF-tGFP was then immunoprecipitated and the phosphorylation state of Ser253 within TF-tGFP was assessed by western blot analysis. Because of immunoprecipitation, ratios were calculated against TF antigen, not a housekeeping gene. Moreover, due to the low yield of immunoprecipitation in HCAEC, only the data from HDBEC have been presented. The suppression of PPT expression reduced the phosphorylation of TF_Wt_-tGFP at Ser253 following the activation of PAR2 compared to cells transfected with control siRNA (Figure 5A). In addition, Ser253 was shown to be phosphorylated in non-activated cells expressing TF_Ser245_-tGFP and further increased following PAR2 activation (Figure 5B,C). In contrast, phosphorylation of Ser253 was significantly lower in cells expressing TF_Phe245_-tGFP, even following PAR2 activation. Similarly, Ser253 appeared to be phosphorylated in non-activated cells expressing TF_Phe241_-tGFP but not in cells expressing TF_Ser242_-tGFP or TF_ShortTM_-tGFP (Figure 5C,D). Ser253-phosphorylation also increased in TF_Phe241_-tGFP expressing cells following activation but was lower in cells expressing TF_Ser242_-tGFP and TF_ShortTM_-tGFP. TF_LongTM_-tGFP was not tested in this study. The levels of TF-tGFP incorporated into released microvesicles also reflected the state of Ser253 phosphorylation but not in the same magnitudes (Figure 5E). Furthermore, the density of released microvesicles was unaltered by the type of TF mutation (Supplementary Figure S3). Finally, to discriminate between the contributions of de-palmitoylation and phosphorylation to the fXa-generation associated with cell surface TF, HDBEC transfected to express TF_Wt_-tGFP, or alternatively, MDA-MB-231 cells were pre-incubated with the PKCα inhibitor Gö6976 (100 nM). Inhibition of PKCα with Gö6976 did not alter fXa-generation in HDBEC expressing TF_Wt_-tGFP (Figure 5F) or in MDA-MB-231 cells (Figure 5G).

### 3.7. Sequential De-Palmitoylation and Phosphorylation of TF Can Co-Ordinate Cell Proliferation and Apoptosis Following PAR2 Activation

**Figure 6:**
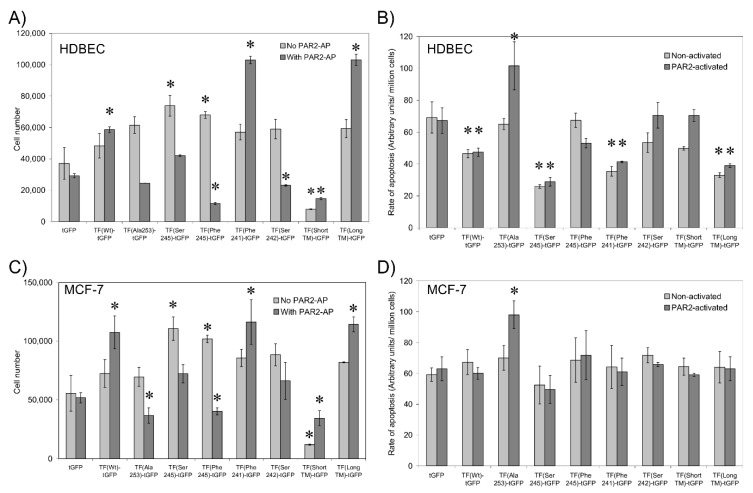
The influence of TF variants on cell proliferation and apoptosis. HDBEC and MCF-7 cells (2.5 × 10^4^) were transfected to express the TF variants and were activated with PAR2-AP (20 µM). The rate of cell proliferation (**A**,**C**) and cell apoptosis (**B**,**D**) were determined using the crystal violet and a chromogenic apoptosis assay, respectively (*n* = 3, * = *p* < 0.05 vs. the respective tGFP-expressing cell sample).

The activation of TF and its interactions with other proteins have been associated with changes in cell proliferation and the induction of apoptosis. To further elucidate the mechanisms by which TF mediates these cellular processes, we examined the change in cell numbers and the level of cellular apoptosis in HDBEC and MCF-7 cells expressing the TF-tGFP variants as above. For comparison, additional samples were transfected to express TF_Ala253_-tGFP to prevent TF phosphorylation. Determination of cell numbers showed increases in cell numbers in both non-activated HDBEC (Figure 6A) and MCF-7 cells (Figure 6C) on expression of TF_Ser245_-tGFP and TF_Phe245_-tGFP compared to cells expressing TF_WT_-tGFP, but a significant reduction in expression of TF_ShortTM_-tGFP. Furthermore, PAR2 activation of the cells resulted in increased proliferation at 24 h in cells expressing TF_Wt_-tGFP, TF_Phe241_-tGFP and TF_LongTM_-tGFP compared to their respective control cells expressing tGFP. However, a reduction in the number of both cell types was observed in PAR2-activated cells expressing TF_Ala253_-tGFP and TF_Phe245_-tGFP and also in HDBEC expressing TF_Ser242_-tGFP compared to their respective control cells expressing tGFP (Figure 6A,C). In agreement with the proliferation data, lower levels of endothelial but not MCF-7 cell apoptosis was detectable in cells expressing TF_Wt_-tGFP, TF_Ser245_-tGFP, TF_Phe241_-tGFP and TF_LongTM_-tGFP, while increased levels of apoptosis were detected in endothelial and MCF-7 cells expressing TF_Ala253_-tGFP at 24 h post-activation (Figure 6B,D).

## 4. Discussion

The ability of fVIIa to activate coagulation is dependent on the formation of a complex with cell-surface TF. However, encrypted TF may form a complex with fVIIa without promoting significant coagulation [3,4,5,6,7,8]. The kinetics of these interactions have previously been studied [3,22,37]. In agreement with this concept, similar amounts of fVIIa-HRP were bound to the surface of HDBEC transfected to express TF before and after activation (Figure 1B and Table 2). Previously, we showed that PAR2 activation of MDA-MB-231 cells resulted in a 56% increase in cell-surface TF antigen at 30 min post-activation [14]. However, endothelial cells exhibit a relatively slower response rate to PAR2 activation compared to MDA-MB-231 cells [13], and also, the 20 min activation time used in this study was even shorter than that previously used for MDA-MB-231 cells [14]. Accordingly, neither the amount of TF-tGFP antigen expressed on the surface of HDBEC (not shown) nor the binding of fVIIa-HRP changed following PAR2 activation (Table 2). Therefore, the increases in fXa-generation observed did not arise from changes in cell-surface TF-tGFP antigen.

Reversible S-palmitoylation of TF was one of the earliest post-translational modifications reported, and it occurs on Cys245 of human TF [18,19]. Currently, palmitoyl-protein thioesterase (PPT) 1 and 2 (also known as acyl-protein thioesterase) are the identified enzymes responsible for the de-palmitoylation of cellular proteins, although controversies regarding the role, location and number of these enzymes do exist [32,38]. These enzymes have been implicated in the regulation of proteins as diverse as eNOS [39], p21RAS [40], PSD-95 [32] and growth-associated protein-43 (GAP-43) [41]. PPT enzymes may be initiated in response to intracellular Ca^2+^ ion release [39] and regulate proteins through reversible de-palmitoylation of cysteine residues [42]. The de-palmitoylation of proteins is a sequence-specific process [32] and determines the trafficking and localisation of proteins within cells [43]. Due to the homology between PPT1 and PPT2, we employed a confirmed siRNA that is suitable for silencing both genes concurrently. The suppression of PPT expression in cells expressing TF_Wt_-tGFP reduced fXa-generation following the activation of the cells without affecting fVIIa-HRP binding (Figure 2 and Table 2). However, knock-down of PPT may have additional unknown effects on cells other than preventing de-palmitoylation of TF. Therefore, in further studies, palmitoylation of TF was prevented by Cys245→Ser substitution. This substitution increased fXa-generation in resting cells (Figure 2D), whereas replacing this residue with phenylalanine to mimic palmitoylation curtailed the increase in TF procoagulant activity following PAR2 activation (Figure 2E) and partially hindered fVIIa binding. Therefore, while the ability of TF to bind fVIIa is not altered by its palmitoylation state, the ability of the complex to generate fXa is significantly amplified following de-palmitoylation of TF. A possible explanation for this enhanced fXa-generation may include the translocation of TF to lipid-raft domains that support TF activity through the exposure of phosphatidylserine on the surface of the cells. This hypothesis is supported by the suggestion that, following cellular activation, TF translocates across membrane domains of differing densities [37,44]. The purpose of the various mutational changes and possible explanations for the main observations have been summarised in Table 4. In fact, TF is released within low-density microvesicles [30], which must accordingly arise from membrane domains with low density [20,45,46]. Mutations in palmitoyl-protein thioesterase are known to lead to neurodegenerative disorders [47]. However, different mutations are thought to result in a broader spectrum of clinical presentations [48,49]. These include influences on the pro-inflammatory and immune responses [50,51], the apoptotic mechanisms [52] and also tumour progression [53]. Therefore, currently any association with blood haemostasis remains tenuous, and we have refrained from speculation. However, the availability of PPT knock-out mice [48,54] may produce novel and interesting connections, particularly regarding the role of TF during the inflammatory responses and/or in cancer progression [55].

**Figure 7:**
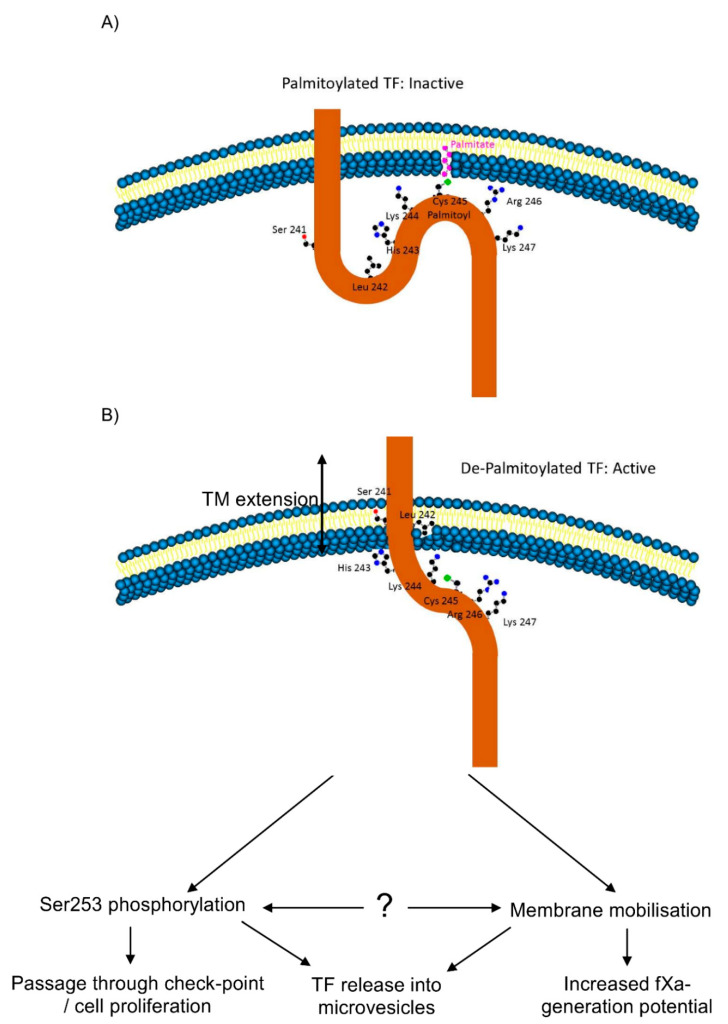
Schematic representation of the outcome of TF de-palmitoylation on the transmembrane and cytoplasmic domains. (**A**) Palmitoylation of Cys245 retains the transmembrane-cytoplasmic interface-region within the cytoplasm. (**B**) De-palmitoylation of TF permits Ser241-Leu242 to be incorporated into the cell membrane elongating the transmembrane domain from 21 to 23 amino acids.

Activation of cells expressing TF_Wt_-tGFP resulted in the aggregation of TF on cells (Figure 3, Table 3). Moreover, prevention of palmitoylation induced spontaneous aggregation of TF_Ser245_-tGFP even in resting cells. In contrast, activation of cells did not result in significant aggregation either in cells expressing TF_Phe245_-tGFP or in cells following suppression of PPT1 and PPT2 expression. These findings are in line with those reporting a lower procoagulant activity associated with palmitoylated TF [11,12,19] arising from decreased association with cell-surface lipid rafts [5]. The translocation/aggregation of TF and the association with lipid rafts following de-palmitoylation also suggests alterations in the orientation of the transmembrane domain. Recently, alternative mechanisms by which palmitoylation can modify receptor protein function have emerged. These are mainly mediated through adjusting the orientation and length of the transmembrane domain [33,36]. Modifications in the length and orientation of the transmembrane domain allow the incorporation of these membrane proteins into different membrane domains with varying membrane thickness. In fact, the transmembrane domain of TF consists of 21 amino acid residues, which is shorter than the average 23 residue length of the integral plasma membrane proteins [56]. Consequently, an increase in the span of the transmembrane domain may permit the transfer of TF into thicker lipid-raft domains [33,34,36] and could result in higher procoagulant activity. The presence of the Ser241-Leu242 di-peptide at the transmembrane-cytoplasmic interface within TF produces a motif of neutral hydrophobicity (Figure 7). This suggests that these two amino acids may remain within the cytoplasm or alternatively be buried within the membrane bilayer, depending on other influences such as palmitoylation of the proximal Cys245 residue. In support of this, increasing the length of the available membrane-spanning region resulted in higher activity in resting cells while shortening, and discouraging the incorporation of the di-peptide into the membrane suppressed the increase in fXa-generation following cellular activation (Figure 4). Interestingly, the ability of the shortened TF variant to bind fVIIa was significantly reduced but only following cellular activation, suggesting a robust cellular attempt to translocate TF to thicker membrane regions, which may in turn disrupt the TF-fVIIa complex due to steric hindrance. Interference with the TF-fVIIa complex was also observed following the expression of TF_Val225_-tGFP to reduce the flexibility at the mid-section of the transmembrane domain, also indicating that the TF–fVIIa interaction could not be accommodated. In previous studies, the expression of truncated forms of TF in cells showed little variation in activity compared to full-length TF [9,10]. However, these studies measured the TF activity in activated/perturbed cells only. Our data explain the results obtained using cytoplasmic domain-deleted TF since the palmitoylation of the cytoplasmic domain appears to be essential for the encryption rather than the decryption of TF.

De-palmitoylation of TF precedes and enhances the rate of TF phosphorylation [11,12]. The subsequent TF phosphorylation at Ser253 is concurrent with increased TF activity [14] and release within cell-derived microvesicles [13]. In agreement with a previous report [11], the lack of TF palmitoylation (TF_Ser245_-tGFP) enhanced TF phosphorylation and release within microvesicles in resting cells (Figure 5). Moreover, encouraging membrane-incorporation of the transmembrane-cytoplasmic interphase domain (TF_Phe241_-tGFP) was equally effective, suggesting that TF phosphorylation at Ser253 is dependent on the length of the transmembrane domain. In contrast, prevention of de-palmitoylation using either PPT siRNA or by mimicking palmitoylation (TF_Phe245_-tGFP) reduced TF phosphorylation at Ser253 and its release within microvesicles. Furthermore, shortening of the transmembrane region by expressing either TF_Ser242_-tGFP or TF_ShortTM_-tGFP equally decreased the level of TF phosphorylation at Ser253 and reduced TF release (Figure 5). It is possible that the lengthening of the transmembrane domain permits the approximation of PKCα to the TF cytoplasmic domain containing Ser253, prior to translocation of TF to lipid-rafts. However, PKCα has been suggested to be associated with lipid-rafts, which explains how the translocation of TF to lipid-rafts may promote TF phosphorylation by PKCα. Moreover, since inhibition of PKCα using Gö6976 did not alter the fXa-generation capacity, our data suggest that the fXa-generation may be independent of TF phosphorylation and release from cells.

The expression of TF_Wt_-tGFP induced cell proliferation in both endothelial cells and the MCF-7 cell line following PAR2-activation (Figure 6). This was concurrent with a reduction in cell apoptosis in endothelial cells but was not significant in the MCF-7 cell line. Intriguingly, the expression of TF_Ser245_-tGFP or TF_Phe245_-tGFP resulted in increased proliferation in resting cells but declined significantly with TF_Phe245_-tGFP, following PAR2 activation. This suggests that de-palmitoylation of TF *per se*, is not a signal for induction of cell proliferation. Additionally, extending the transmembrane domain (TF_Phe241_-tGFP or TF_LongTM_-tGFP) was highly proliferative following PAR2 activation, while reduced rates of proliferation were associated with the expression of the shorter TF_Ser242_-tGFP and TF_ShortTM_-tGFP. Therefore, the induction of cellular proliferation signalling appears to involve the translocation of TF within the cell membrane and is complemented with a signal arising from PAR2 activation [13]. The expression of TF_Wt_-tGFP, TF_Ser245_-tGFP, TF_Phe241_-tGFP or TF_LongTM_-tGFP also reduced the rate of cell apoptosis in HDBEC but not MCF-7 cells (Figure 6). However, the rate of apoptosis did not match the reduction in cell proliferation observed in cells expressing TF_Phe245_-tGFP, TF_Ser242_-tGFP and TF_ShortTM_-tGFP. Together, these data suggest that the induction of apoptosis is likely to be associated with the lack of phosphorylation of Ser253, and therefore, the impairment in the release of excess TF from cells and may hence be distinct from the processes that induce proliferation [28,57,58].

## 5. Conclusions

In conclusion, this study indicates that the procoagulant activity of TF may be enhanced by the palmitoylation-dependent modifications in the orientation of the transmembrane domain of TF and is likely to mobilise TF to particular regions within the cell membrane. Furthermore, the increase in the procoagulant activity is initiated by de-palmitoylation and appears to be independent of TF phosphorylation. Finally, following cellular activation, separate proliferative and pro-apoptotic TF signals are initiated through phosphorylation-independent and -dependent mechanisms, respectively. At least some of these mechanisms appear to be independent of the procoagulant function of TF and may therefore determine the level of damage and the fate of the cells following injury or trauma.

## Figures and Tables

**Table 1:** The list of TF variants and DNA primers used for mutagenesis.

Plasmid	Mutation	Primer Set
pCMV-Ac-TF_Ser245_-tGFP	Cys245→Ser	5′-TCTACACAAGAGTAGAAAGGCAG
		5′-GATATAGCCAGGATGATG
pCMV-Ac-TF_Phe245_-tGFP	Cys245→Phe	5′-CTACACAAGTTTAGAAAGGCAG
		5′-AGATATAGCCAGGATGATG
pCMV-Ac-TF_Phe241_-tGFP	Ser241→Phe	5′-GTGGCTATATTTCTACACAAGTG
		5′GATGATGACAAGGATGATG
pCMV-Ac-TF_Ser242_-tGFP	Leu242→Ser	5′-GGCTATATCTTCACACAAGTGTAG
		5′-AGGATGATGACAAGGATG
pCMV-Ac-TF_ShortTM_-tGFP	Ser241-Leu242-del	5′-CACAAGTGTAGAAAGGCAG
		5′-TATAGCCAGGATGATGAC
pCMV-Ac-TF_LongTM_-tGFP	Ser243-Leu244-add	5′-TCCCTGCACAAGTGTAGAAAGGCAG
		5′-TAGAGATATAGCCAGGATG
pCMV-Ac-TF_Val225_-tGFP	Gly225→Val	5′-TACATCATTGTAGCTGTGGTATTTG
		5′-GAATATTTCTCTGAATTCCC

**Table 2:** Analysis of the interaction of fVIIa-HRP with TF-tGFP variants expressed on the cell surface.

TF Variant	Non-Activated (Fmol/Million Cells)	PAR2-Activated (Fmol/Million Cells)
tGFP	11.81 ± 1.19 *	10.2 ± 1.28 *
TF_Wt_-tGFP	20.15 ± 2.56	20.7 ± 2.33
TF_Ser245_-tGFP + PPT siRNA	19.33 ± 1.03	19.51 ± 0.92
TF_Ser245_-tGFP + control siRNA	19.70 ± 0.64	20.01 ± 0.38
TF_Ser245_-tGFP	18.29 ± 2.12	19.6 ± 0.99
TF_Phe245_-tGFP	17.04 ± 1.59	14.4 ± 1.01 *
TF_ShortTM_-tGFP	18.13 ± 4.04	8.8 ± 2.87 *
TF_LongTM_-tGFP	20.80 ± 1.50	21.0 ± 1.51
TF_Phe241_-tGFP	20.00 ± 1.00	20.0 ± 0.82
TF_Ser242_-tGFP	20.00 ± 2.00	19.0 ± 2.50
TF_Val225_-tGFP	17.00 ± 2.05	12.0 ± 3.10 *

HDBEC were co-transfected to express TF_Wt_-tGFP with PPT or control siRNA. Alternatively, cells were transfected to express the TF variants as indicated in the table as well as a control set expressing tGFP. The cells were incubated with fVIIa-HRP for 10 min and washed, and the amount of bound fVII-HRP was determined against a standard curve. (*n* = 4, * = *p* < 0.05 vs. cells expressing TF_Wt_-tGFP.)

**Table 3:** Estimation of TF aggregate sizes.

Sample	Non-Activated (µm)	PAR2-Activated (µm)
tGFP	0.09 ± 0.03	0.09 ± 0.03
TF_Wt_-tGFP	0.15 ± 0.06	0.43 ± 0.11
TF_Ser245_-tGFP	0.41 ± 0.10	0.53 ± 0.12
TF_Phe245_-tGFP	0.09 ± 0.03	0.12 ± 0.05
TF_Wt_-tGFP +PPT siRNA	0.12 ± 0.04	0.12 ± 0.04

HDBEC (5 × 10^4^) were transfected to express TF_Wt_-tGFP in the presence and absence of PPT-siRNA or to express TF_Ser245_-tGFP or TF_Phe245_-tGFP as in Figure 3. Sets of cells were then activated for 20 min using PAR2-AP (20 µM), and the distribution of TF-tGFP was examined. Analysis of the aggregate sizes was carried out using the ImagePro Plus software.

**Table 4:** Summary of the purpose of TF modifications and the main observation.

Plasmid	Purpose	Observation	Possible Explanation
pCMV-Ac-TF_Ser245_-tGFP	Prevent palmitoylation	↑TF activation	TF can mobilise within the membrane
		↑Proliferation	
		(non-activated)	
pCMV-Ac-TF_Phe245_-tGFP	Mimic palmitoylation	↓TF activation	TF cannot mobilise or be released–Increased membrane thickness hampers fVIIa binding
		↓Proliferation	
		↓fVIIa binding	
		(activated)	
pCMV-Ac-TF_Phe241_-tGFP	Increase hydrophobicity of TMD	↑TF activation	Facilitates mobilisation to thicker membrane regions
		↑Proliferation	
		(activated)	
pCMV-Ac-TF_Ser242_-tGFP	Decrease hydrophobicity of TMD	↓TF activation	Hampers mobilisation to thicker membrane regions
		↓Proliferation	
		(activated)	
pCMV-Ac-TF_ShortTM_-tGFP	Shorten TMD	↓TF activation	Cannot mobilise to to thicker membrane regions
		↓Proliferation	
		↓fVIIa binding	
pCMV-Ac-TF_LongTM_-tGFP	Lengthen TMD	↑TF activation	Facilitates mobilisation to thicker membrane regions
		↑Proliferation	
		(activated)	
pCMV-Ac-TF_Val225_-tGFP	Reduce TMD flexibility	↓TF activation	Cannot accommodate correct structure for interaction with fVIIa
		↓fVIIa binding	

## Data Availability

Not applicable.

## Supplementary Materials

The following are available online at https://www.mdpi.com/article/10.3390/cancers13153837/s1, Figure S1: Confirmation of fVIIa-HRP activity and binding to TF, Figure S2: Analysis of the association of TF and PAR2 in the presence and absence of fVIIa, examined in HCAEC, Figure S3: Quantification of HDBEC-derived microvesicles using the Zymuphen Assay l.
