# Supplementary material for: De-Palmitoylation of Tissue Factor Regulates Its Activity, Phosphorylation and Cellular Functions
Source: Cancers (Basel). 2021 Jul 30;13(15):3837. doi: 10.3390/cancers13153837 (PMC8345185; doi:10.3390/cancers13153837)
Supplement: Supplementary file 1 [file cancers-13-03837-s001.zip › cancers-1280942-supplementary.pdf]

De-palmitoylation of tissue factor regulates its activity, phosphorylation and cellular functions

Camille Ettelaie[1*], Sophie Featherby[1], Araci M R Rondon[2], John Greenman[1], Henri H Versteeg[2], Anthony Maraveyas[3]

Running title: Regulation of TF function by palmitoylation

[1]Biomedical Section, University of Hull, Cottingham Road, Hull, HU6 7RX, UK. UK, [2]Einthoven Laboratory for Vascular and Regenerative Medicine, Division of Thrombosis and Hemostasis, Department of Internal Medicine, Leiden University Medical Center, Leiden, The Netherlands [3]Division of Cancer-Hull York Medical School, University of Hull, Cottingham Road, Hull, HU6 7RX, UK.

*Correspondence to Dr Camille Ettelaie, Biomedical Section, Department of Biological Sciences, University of Hull, Cottingham Road, Hull, HU6 7RX, UK

Email: C.Ettelaie@hull.ac.uk

Tel: +44(0)1482-465528

Fax: +44(0)1482-465458

ORCID number: 0000-0002-6121-5262

Supplementary Figure S1 Confirmation of fVIIa-HRP activity and binding to TF

A)

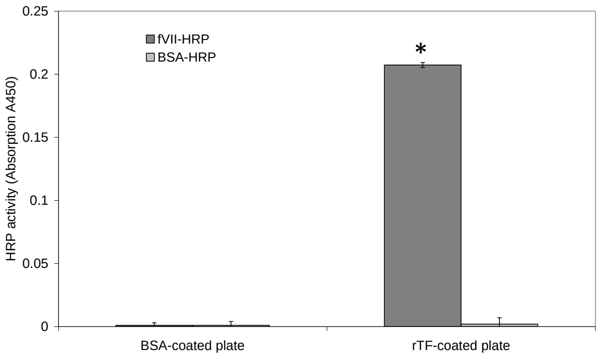

B)

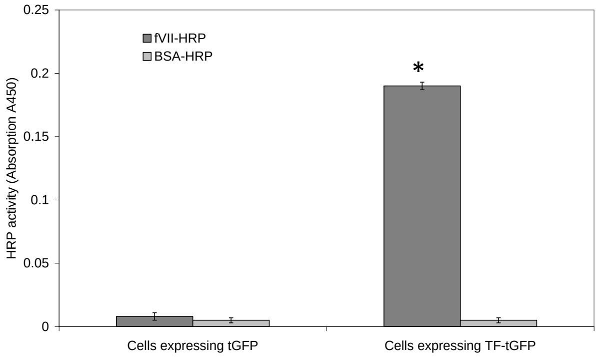

C)

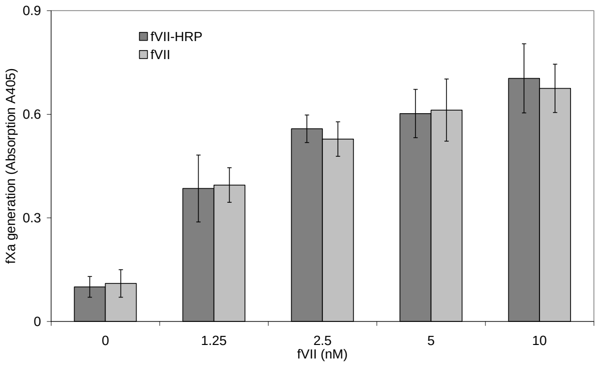

Factor VIIa-HRP and BSA-HRP conjugates were prepared using the Lightning-link HRP kit. Samples (20 nM) were incubated for 15 min in A) 48-well plates pre-coated with recombinant Innovin TF (13 ng/ml) in BSA (1 % w/v) or the vehicle solution, or B) with transfected-HDBEC expressing either TF-tGFP or tGFP. The plates were washed with PBS and the HRP activity determined using the One-solution TMB substrate (200 µl). (n= 3, * = $p<0.05$ vs. BSA-HRP sample). C) Equal amounts of fVIIa-HRP and unconjugated-fVIIa (0-10 nM) were added to HDBEC expressing TF-tGFP and incubated for 10 min following which fXa-generation was measured. (n= 5).

Supplementary Figure S2 Analysis of the association of TF and PAR2 in the presence and absence of fVIIa, examined in HCAEC

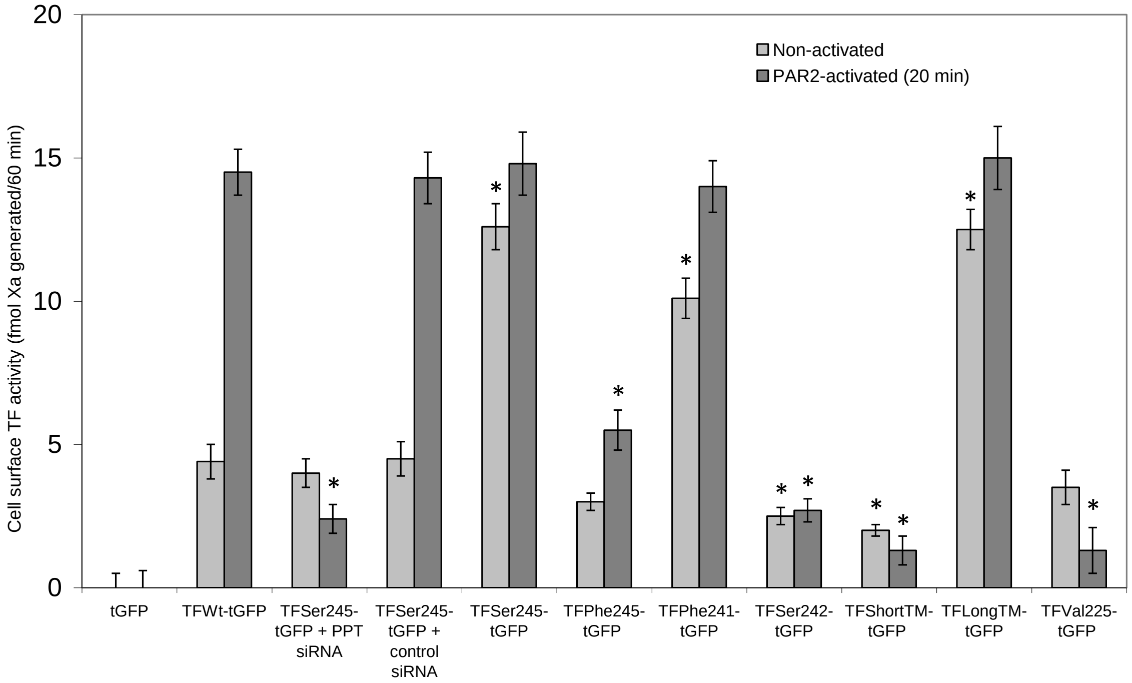

Human coronary artery endothelial cells (HCAEC; 5×10^4) were co-transfected with combinations of pCMV-Ac-TF-tGFP and PPT-siRNA or control siRNA. HCAEC (5×10^4) were also transfected to express TF_{Wt}-tGFP, TF_{Ser245}-tGFP, TF_{Phe245}-tGFP $TF_{ShortTM}$-tGFP, TF_{LongTM}-tGFP, TF_{Phe241}-tGFP, TF_{Ser242}-tGFP or TF_{Val225}-tGFP. Sets of cells were activated using PAR2-agonsit peptide and fXa-generation was measured after 20 min. (n= 3, * = p<0.05 vs. the respective cells expressing TF_{Wt}-tGFP).

Supplementary Figure S3 Quantification of HDBEC-derived microvesicles using the Zymuphen Assay

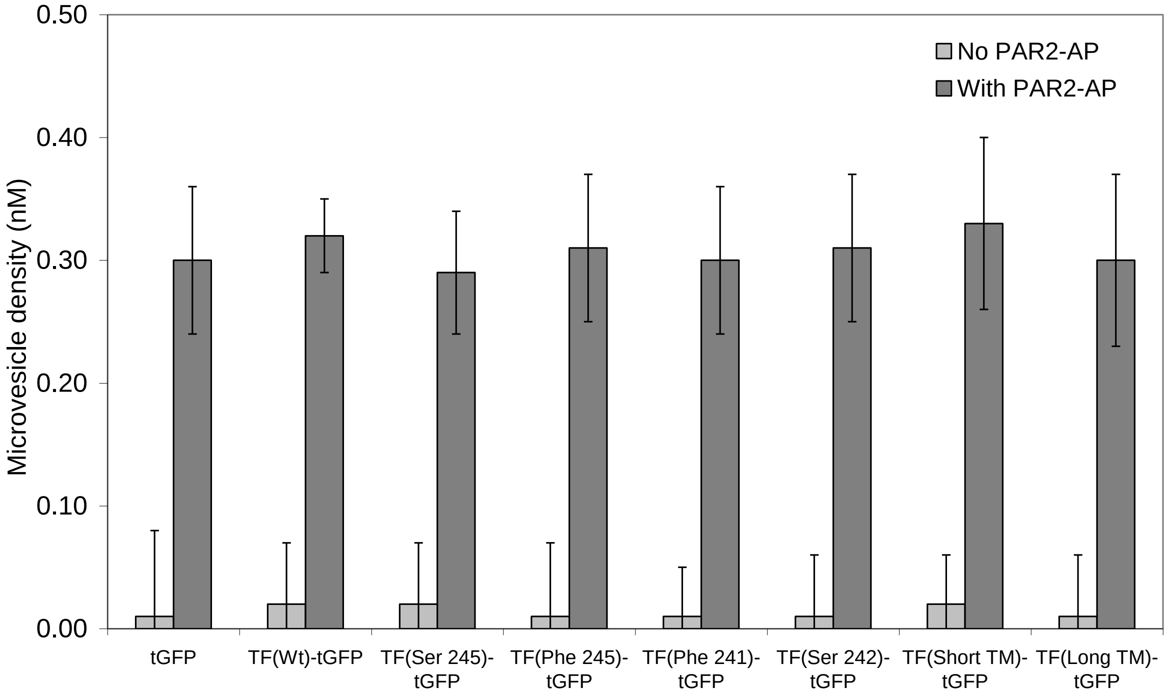

HDBEC (5×10^4) were transfected with the TF variants as shown and one set of cells were activated using PAR2-AP (20 µM). The density of released microvesicles was then measured using the Zymuphen Microparticle Assay Kit. (n= 4).
